# Influence of Fast Drying on the Morphology of α-Fe_2_O_3_ and FeMnO_3_/α-Fe_2_O_3_ Fibers Produced by Solution Blow Spinning

**DOI:** 10.3390/nano14030304

**Published:** 2024-02-02

**Authors:** Lara Nágela Lopes Cavalcante Barros, Rondinele Nunes de Araujo, Emanuel Pereira do Nascimento, Alexandre José de Almeida Gama, Gelmires Araújo Neves, Marco Antonio Morales Torres, Romualdo Rodrigues Menezes

**Affiliations:** 1 Graduate Program in Materials Science and Engineering, Federal University of Campina Grande, Campina Grande 58429-900, Brazil; lara.nagela@estudante.ufcg.edu.br (L.N.L.C.B.); emanuel.nascimento@tecnico.ufcg.edu.br (E.P.d.N.); 2 Laboratory of Materials Technology, Federal University of Campina Grande, Campina Grande 58840-000, Brazil; agama@df.ufcg.edu.br (A.J.d.A.G.); gelmires.neves@ufcg.edu.br (G.A.N.); 3 Department of Theoretical and Experimental Physics, Federal University of Rio Grande Do Norte, Natal 59078-970, Brazil; marco.morales@ufrn.br

**Keywords:** fibers, fast drying, A-HSBS, FeMnO_3_/α-Fe_2_O_3_

## Abstract

α-Fe_2_O_3_ and FeMnO_3_/α-Fe_2_O_3_ fibers were successfully prepared via Solution Blow Spinning (SBS). The effect of drying during the SBS process on fiber morphology was evaluated by scanning electron microscopy (SEM), transmission electron microscopy (TEM), and N_2_ adsorption–desorption isotherms. A slow drying promoted continuous fibers with rough surfaces and lower average diameters. However, fast drying enabled the production of fibers with low densification and many surface pores with higher BET-specific surface areas. The porous fibers produced have potential applications in energy generation and storage.

## 1. Introduction

Materials with micro, sub-metric, and nanofibrillar morphologies have gained attention in several applications due to their high surface/volume ratio and surface reactivity [1]. Electrospinning is the main technique studied in the production of nanofibers. However, it has several disadvantages, such as requiring an excessively high power supply (up to 60 kV), limitations in solvent types, and low production rates [2]. The Solution Blow Spinning (SBS) technique, which uses an aerodynamic driving force in fiber production, has emerged as a more advantageous process, with higher productivity rates and greater flexibility in the possible solvents and inorganic precursors [3,4].

After the report of Medeiros et al. in 2009 [3], numerous studies focused on using the SBS process to produce polymeric fibers [5,6,7]. However, iron-based SBS fibers were first reported by Farias and collaborators [8], producing spinel-type ferrite nanofibers. Silva and collaborators [9] produced ferrite nanofibers of the MFe_2_O_4_ type (M = Cu, Co, Ni), and then Raimundo and collaborators manufactured NiFe–NiFe_2_O_4_ composite nanofibers [10].

Authors often seek to use highly volatile organic solvents, such as dimethyl sulfoxide (DMSO) [11], chloroform [12], and dimethylformamide (DMF) [13], to facilitate the drying and solidification of fibers during the SBS process. However, these solvents are toxic to human beings and the environment. On the other hand, using low-volatile solvents generates wet fibers in the collector, collapsing the fibrillar morphology through the entanglement of polymer chains [14].

Aiming to use green solvents and overcome the drying difficulties during processing and the consequences of inadequate drying on fiber integrity, previous studies [15] used a slow heating system during SBS to ensure the integrity of fibers generated in the spinning process.

However, recently, Silva and collaborators [16] reported a new SBS apparatus, a modification of the technique denoted by air-heated solution blow spinning, A-HSBS, where a heat gun was introduced in the system, improving and accelerating the drying process of fibers along the work distance and making fiber production possible even using low-volatility solvents such as water and ethanol.

There are only a few studies in the literature that have used SBS to produce ceramic oxide fibers based on iron nitrate and no research using manganese nitrate, despite the great potential of iron–manganese-based systems for energy generation and storage. Only a handful of studies in the literature have used SBS to produce ceramic oxide fibers based on iron nitrate. However, despite the great potential of iron–manganese-based systems for energy generation and storage, research has yet to report the use of manganese nitrate for fiber production. These systems face difficulties in forming and preserving fiber morphology due to the coalescence process’s consequences caused by fiber drying problems. Nitrates, notably manganese nitrate, are very hydroscopic, and this is a challenge when using water as a solvent for the spinning process. This study presents the first-time preparation of α-Fe_2_O_3_ (hematite) and FeMnO_3_/α-Fe_2_O_3_ fibers using the Solution Blow Spinning technique. Moreover, we have conducted an evaluation of the influence of the drying rate in the SBS process on the fiber morphology of the fibers using a slow-drying system and a fast-drying apparatus (A-HSBS).

## 2. Materials and Methods

### 2.1. Materials

The following chemicals were used in the study: iron nitrate nonahydrate [Fe(NO_3_)_3_·9H_2_O, Sigma Aldrich, St. Louis, MI, USA, 98%], manganese nitrate tetrahydrate [Mn(NO_3_)_2_·4H_2_O, NEON, Suzano, SP, Brazil, 98%], polyvinylpyrrolidone (PVP, MW = 1,300,000 g/mol, Sigma Aldrich), and acetic acid (C_2_H_4_O_2_, Dinamica, Indaiatuba, SP, Brazil, 99.7%, 17.4 mol/L).

### 2.2. Fiber Production

**Figure 1:**
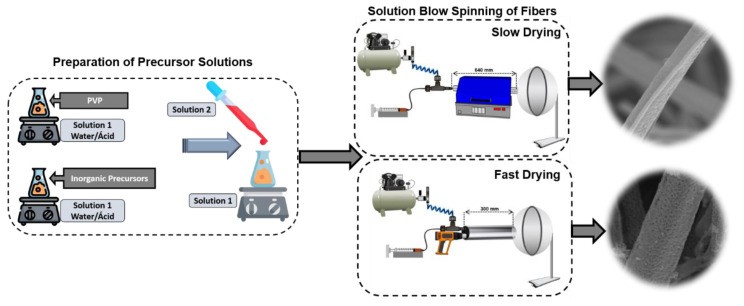
Schematic diagram of the Solution Blow Spinning process used to obtain fibers.

The precursor solutions were prepared using a methodology adapted from previous research [15]. Two solutions were prepared separately, as shown in Figure 1. The first solution (solution 1) was prepared as follows: 12% (*m*/*v*) of PVP was added to 0.5 mL of water and 3.5 mL of acetic acid (final concentration of the acid solution = 15.3 mol/L), remaining under vigorous stirring for one hour. Then, the second solution (solution 2) was produced: to obtain pure iron, 1.92 g of iron nitrate was dissolved in 0.5 mL of water and 3.5 mL of acetic acid; for the Fe/Mn system, the nitrates were dissolved in a molar ratio of Fe:Mn = 1:1. Once solubilization had taken place, the solutions containing the inorganic precursors (solution 2) were added to the solution containing PVP (solution 1) under vigorous magnetic stirring for two hours.

The solutions underwent the Solution Blow Spinning (SBS) process. The impact of slow and fast drying/heating modes on fiber morphology was evaluated during fiber spinning using two SBS apparatuses (Figure 1). The slow-drying SBS apparatus is composed of a compressed air source, injection pump, SBS nozzle (set of concentric nozzles), heated working distance constituted by a tubular oven (EDG, model FT20HI), with an interior temperature of 100 °C, and static collector.

Conversely, for fast heating, a heat gun (DeWalt, model D26411) is placed parallel to the spinning nozzle, and the working distance is delimited by the length of a steel tube (Figure 1). Inside the tube, the temperature was approximately 100 °C. All spinning parameters were kept constant in both procedures: air pressure of 1.5 bar, injection rate of 5 mL/h, and protrusion distance of 15 mm. The working distance was 640 mm for the tubular furnace and 300 mm for the fast-drying system. The iron oxide fibers were calcined under an air atmosphere at 800 °C with a rate of 2 °C/min and a dwelling time of 120 min, and the Fe:Mn = 1:1 fibers were calcined under an air atmosphere at 800 °C and 1000 °C with a rate of 2 °C/min and a dwelling time of 120 min.

### 2.3. Characterization

Fiber morphology was analyzed using scanning electron microscopy (SEM, VEGA 4, TESCAN, Brno, Czechia) and transmission electron microscopy (TEM, JEM-2100, Jeol, Tokyo, Japan). The diameters of approximately 100 fibers were measured using image analysis software (Image J (https://imagej.net/nih-image/ accessed on 29 December 2023), National Institutes of Health, USA). Statistical analyses (t and Mann–Whitney tests) were performed at a 0.05 significance level. The specific surface area was studied using N_2_ adsorption–desorption isotherms. Brunauer–Emmett–Teller (BET) theory was used to determine the surface area, and the Barrett–Joyner–Halenda (BJH) method was applied to determine pore size (Quantachrome ASiQwin, Nova 3200, Boynton Beach, FL, USA). X-ray diffraction was performed (XRD-6000, SHIMADZU, Kyoto, Japan) with Cu Kα radiation, scanning from 10° to 80° with a step of 0.02° and a fixed time mode. Phase determinations of calcined samples were performed by comparing the diffraction patterns with the ICSD standards.

## 3. Results and Discussion

**Figure 2:**
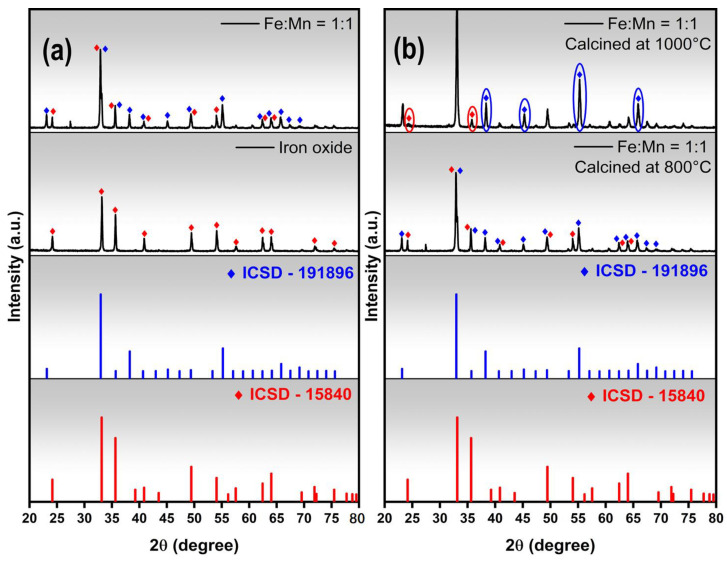
XRD patterns of fibers obtained by fast drying and calcined at 800 °C (**a**) and comparison of the XRD patterns of Fe:Mn = 1:1 fibers calcined at 800 °C and 1000 °C (**b**).

Figure 2 illustrates the crystallographic information of the fibers produced by fast drying and calcining at 800 °C. The iron oxide fibers displayed peaks indicative of the α-Fe_2_O_3_ (hematite) phase (ICSD-15840), possessing a trigonal structure and space group R3c, with no other impurities such as Fe_3_O_4_ (Figure 2a).

The fibers with a Fe:Mn ratio of 1:1 are composed of two phases: α-Fe_2_O_3_ (ICSD-15840) and FeMnO_3_ (ICSD-191896) (Figure 2a). The FeMnO_3_ phase is formed by replacing manganese in the Mn_2_O_3_ structure with iron atoms while maintaining its crystal structure [17]. The presence of the α-Fe_2_O_3_ secondary phase is likely due to the calcination temperature used in this study, which was 800 °C for 2 h. This temperature was insufficient for the complete reaction and formation of pure FeMnO_3_ in the material.

After calcining the Fe:Mn = 1:1 fibers at 1000 °C for two hours (Figure 2b), there was a decrease in the intensity of the characteristic peaks of the α-Fe_2_O_3_ phase, located at 2θ positions of 24.1° and 35.7° (circled in red), and an increase in the intensity of the peaks of the FeMnO_3_ phase located at 2θ positions of 38.3°, 45.2°, 55.2°, and 65.8° (circled in blue). Although an incomplete process, the results indicate a reaction inclination to form the FeMnO_3_ phase, increasing its content. Phase diagrams for the iron(III) oxide and manganese(III) oxide systems are scarce in the literature and have been improved in recent years. It is possible to observe some variations in the diagrams depending on the type of precursor and the synthesis method, which makes it somewhat difficult to analyze and apply the phase diagrams of this system for a more in-depth analysis of the formation behavior of the observed phases. However, by analyzing some works in the literature, it was observed that with intermediate compositions between iron and manganese in the iron oxide/manganese oxide diagram [18], there is a mixture of phases at 800 °C and a single-phase material at 1000 °C. In the diagram presented in another study [19], at 800 °C, the material is single-phased, and at 1000 °C, it shows a phase change to spinel.

In systems where the ions are initially dispersed in polymeric matrices, it was observed [20] that the presence of the polymer can hinder the formation of a single-phase material, which can be explained by the difference in diffusivity of Fe and Mn, generating phase segregation during the thermal process. Yao and collaborators [21] used a 1:1 iron/manganese ratio and found that the FeMnO_3_ phase formed after calcination at 1000 °C. However, when calcined at 800 °C, the same composition resulted in a biphasic system with FeMnO_3_ as the primary phase and α-Fe_2_O_3_ as the secondary phase.

**Figure 3:**
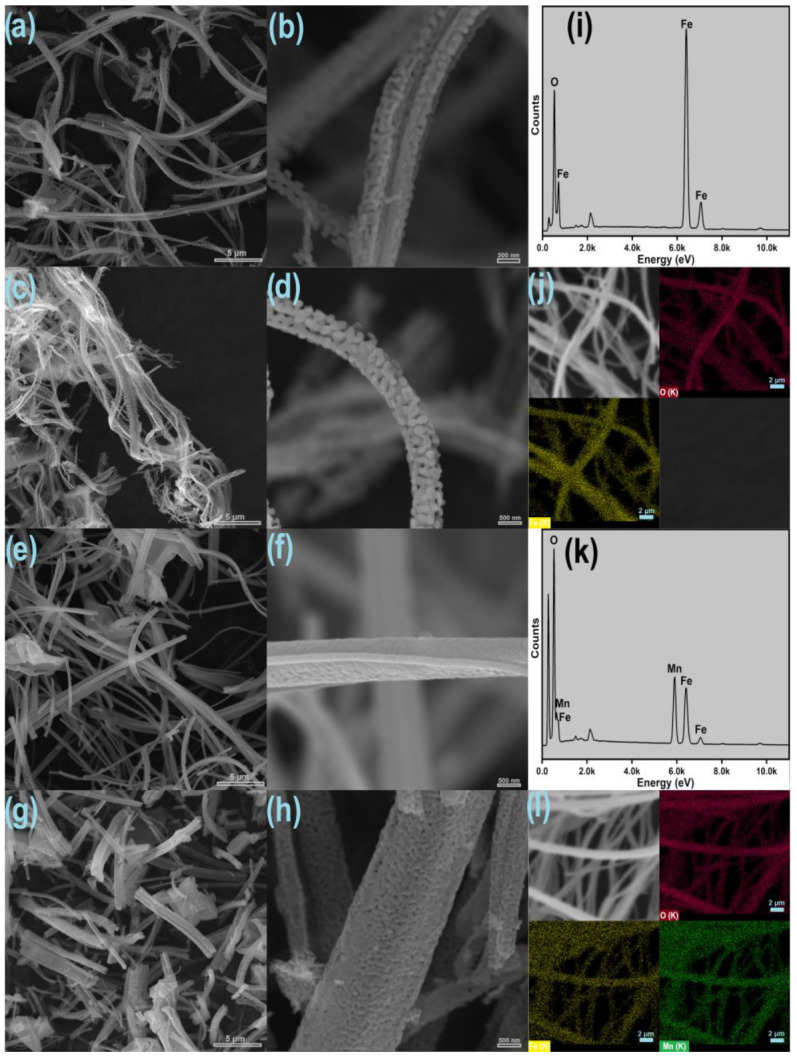
SEM images of pure iron oxide fibers using slow drying (**a**,**b**), fast drying (**c**,**d**), Fe:Mn = 1:1 fibers using slow drying (**e**,**f**), and fast drying (**g**,**h**). EDS spectrum and elemental mapping in iron oxide fibers (**i**,**j**) and Fe:Mn fibers = 1:1 (**k**,**l**) obtained by fast drying.

The morphology of iron oxide and Fe:Mn = 1:1 fibers produced by SBS after calcination at 800 °C are shown in Figure 3. The fibers produced using slow drying (Figure 3a,b,e,f) exhibited a high aspect ratio (length/diameter), a continuous and randomly arranged structure, and longitudinal lengths reaching the tens of microns.

Fibers produced using the fast-drying process are fragmented, as shown in Figure 3c,d,g,h. This morphology is associated with turbulence caused during the convergence of the internal airflow of the spinning nozzle with the hot air from the heat gun, which causes the fibers to break during the stretching process. However, these fibers exhibit a high number of irregular surface pores.

The high hygroscopicity of manganese nitrate compared to iron nitrate favored the coalescence of manganese-containing fibers when using slow drying. This effect of the high hygroscopicity of manganese nitrate is severe. Until now, no manganese oxide or manganese-containing fibers produced by SBS have been observed in the literature.

Silva and collaborators [22] reported the preparation of fibers from hydrated nitrates, highlighting the challenges of incomplete solvent evaporation, resulting in fiber fusion on the collector. Similarly, Cena and collaborators [23] found that systems with low inorganic precursor concentrations and high solvent quantities also have difficult fiber formation, leading to fusion and coalescence at the contact points. These reports corroborate the difficulty we observed in processing hygroscopic nitrates and further support the efficient route developed in this work to produce Fe:Mn-based fibers.

EDS microanalysis and elemental mapping confirm the homogeneous distribution of iron and manganese in the produced fibers (Figure 3i–l). The Fe:Mn = 1:1 sample exhibited good dispersion of Fe/Mn along the fibers, showing atomic fractions of approximately 21.7% of Fe and 19.6% of Mn, approximate values for the 1:1 ratio.

**Figure 4:**
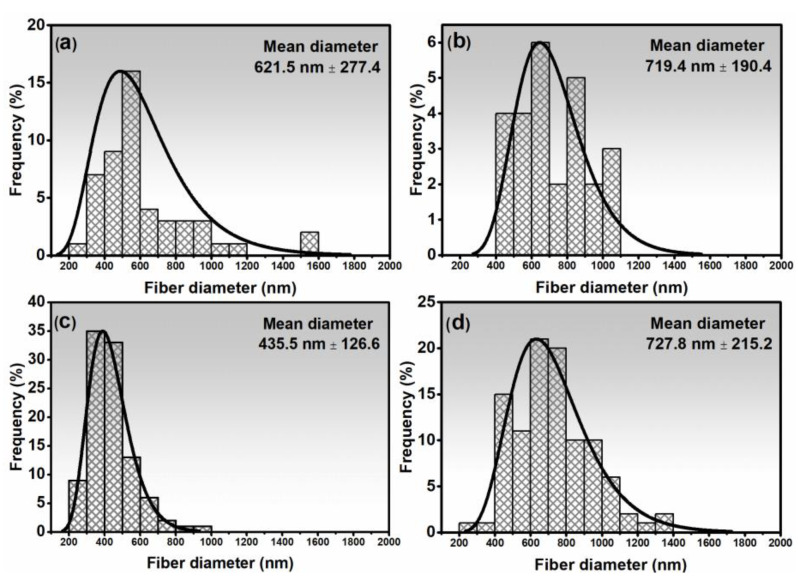
Distribution graphs of the diameters of iron oxide fibers using slow drying (**a**), fast drying (**b**), Fe:Mn = 1:1 fibers by slow drying (**c**), and fast drying (**d**).

Figure 4 shows how the drying and working distance variables affect the average diameter of fibers. Table 1 displays the *p*-values obtained from the Mann–Whitney test. The fibers that underwent fast drying showed significant differences in their distribution curves compared to those obtained through slow drying, as evidenced by their higher average diameters and *p*-values below the 0.05 significance threshold. The fibers produced by rapid drying did not show statistical differentiation in their diameters (Mann–Whitney test, *p* = 0.921). It suggests a possible characteristic of this process: being capable of producing fibers of different compositions with low variability in their diameter distributions.

The fibers produced with a Fe:Mn ratio of 1:1 and slow drying had a narrower range of diameters, between 224.7 nm and 952.4 nm, with an average of 435.5 ± 126.6 nm lower than the other fibers. On the other hand, the same composition produced with rapid drying had a wider diameter distribution, ranging from 1348.7 nm to 201.9 nm with an average of 727.8 ± 215.2 nm.

The rapid drying, favored by the convection currents and rapid heat transfer caused by the heat gun, leads to very rapid evaporation of the solvent on the surface and near-surface regions of the fiber, which causes rapid solidification of the fibers. This process “freezes” the shape of the fiber, preventing the polymer chains from coming together and reducing the diameter of the fibers (caused by the capillary tension of the solvent) as the solvent evaporates. Thus, over time, the solvent migrates and evaporates. However, as the structure has already been formed quickly, the solvent leaves and causes porosity as it leaves but cannot bring the polymer chains closer together. In slow drying, the fiber’s surface is wet (with solvent) for a longer period, allowing for gradual diffusion during drying. There is, therefore, a gradual approximation of the polymer chains caused by the capillary tension of the polymer as the solvent evaporates and binds the chains together, generating shrinkage and a decrease in fiber diameter (and also less porosity in the final fiber). 

According to Jiang and collaborators [20], increasing the amount of Mn precursor in the electrospinning process led to an increase in the diameter of Mn–Fe fibers from 270 nm to 640 nm. The increase in diameter was attributed to the formation of concentric tube-like structures, which made fibers shrink and densify during heat treatment more difficult. In another study by Samuel and collaborators [24], manganese/iron oxide/carbon nanofibers produced using electrospinning and slow drying reached mean diameters up to 688 nm, similar to those obtained in this study. However, Samuel and collaborators [20] used carbon-based fibers and N,N-dimethylformamide (DMF), which is a highly volatile and toxic solvent. These findings suggest that producing Fe/Mn fibers with low diameters is challenging and consistent with previous research.

**Figure 5:**
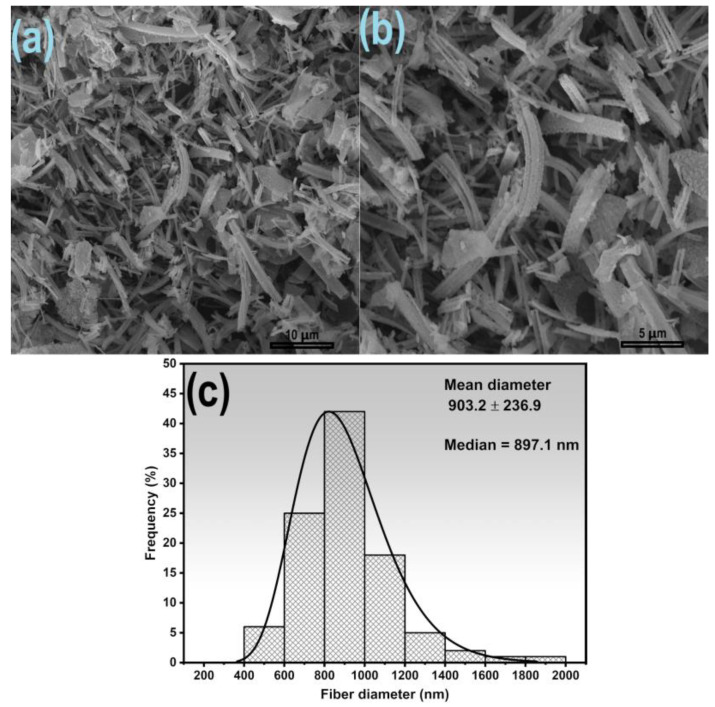
SEM images of Fe:Mn = 1:1 fibers by fast drying after calcination at 1000 °C (**a**,**b**), and distribution graph of the diameters (**c**).

After calcination at 1000 °C, the fast-dried Fe:Mn = 1:1 fibers displayed higher fragmentation, as observed in Figure 5, indicating that the thermal process contributed to the collapse of the fibers. Their diameters varied from 518.2 nm to 1823 nm and an average diameter of 903.2 ± 236.9 nm (Figure 5b), higher than Fe:Mn = 1:1 fibers calcined at 800 °C (Mann–Whitney test, *p* < 0.001).

The fiber sintering process reduced the surface area due to grain growth, resulting in fiber densification and mesopore reduction [25]. As reported by Bhagwan and collaborators [26], excessive calcination at 900 °C resulted in a low BET surface area in CdMn_2_O_4_ fibers. Therefore, calcination at 1000 °C caused damage to fiber morphology and was insufficient to promote the formation of the pure FeMnO_3_ phase. We decided to continue the study with fibers calcined at 800 °C.

**Figure 6:**
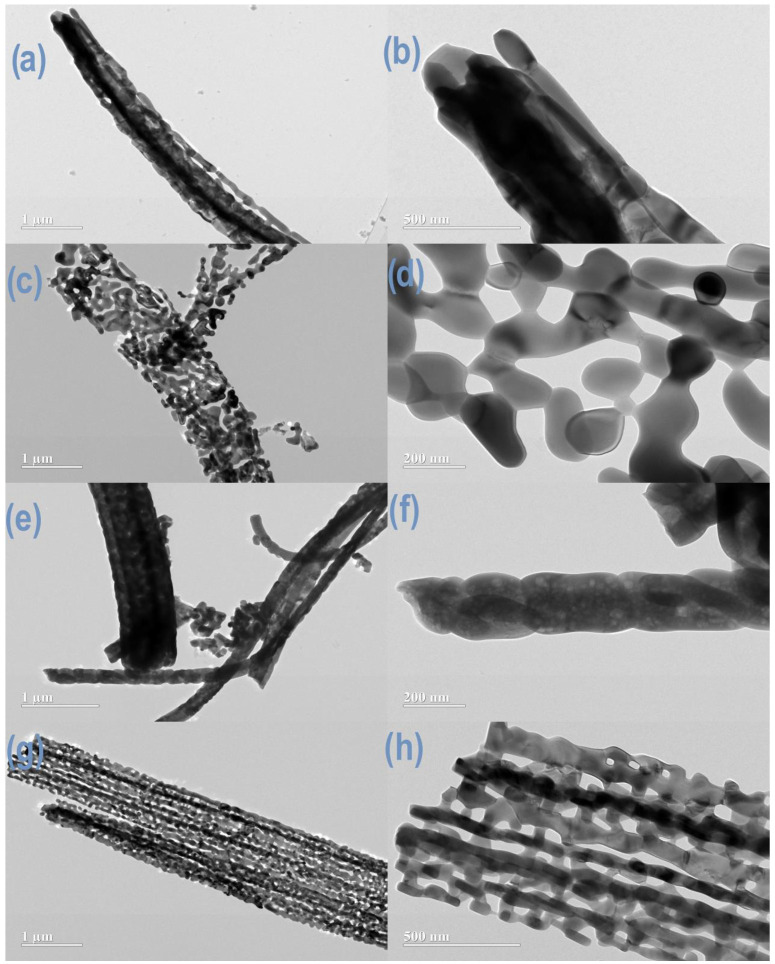
TEM images of pure iron oxide fibers using slow drying (**a**,**b**) and fast drying (**c**,**d**). Fe:Mn = 1:1 fibers using slow drying (**e**,**f**) and fast drying (**g**,**h**).

The structure of the fibers was investigated using transmission electron microscopy (TEM) and illustrated in Figure 6. The results showed that the drying method used impacted fiber morphology. A slower drying process led to higher fiber shrinkage and material coalescence, resulting in dense fibers following the calcination process. It is important to mention that even when drying occurred at a slower solvent evaporation rate using a tubular oven, the dry fibers’ surface remained rough, as depicted in Figure 6a,b,e,f.

The rapid surface solidification caused by the high solvent evaporation rate in the fast-drying process resulted in fibers with regions containing high porosity after calcination (Figure 6c,d,g,h). The fibrillar structure of the iron–manganese-based fibers was preserved by hot air flow parallel to the spinning flow, obtaining a microporous morphology. These findings depict the importance of using heated working distances in SBS apparatus, especially when producing fibers using highly hygroscopic materials.

**Figure 7:**
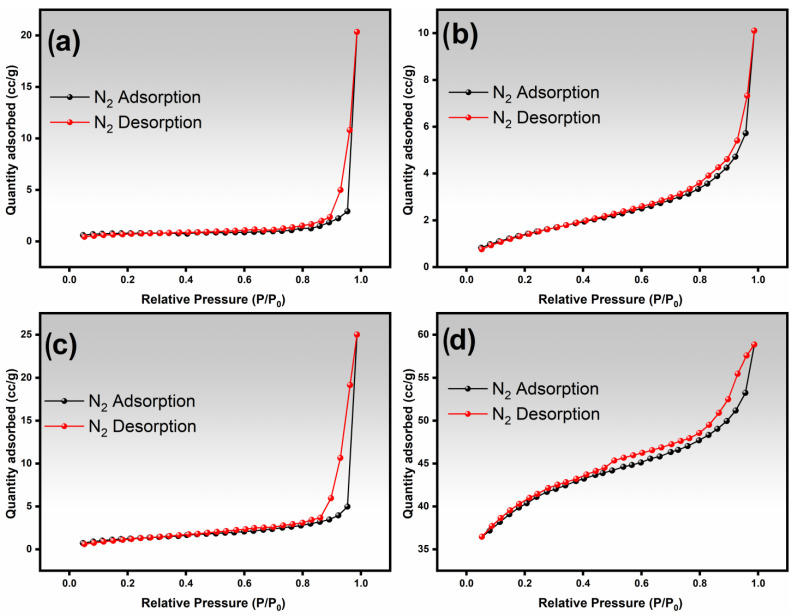
N_2_ adsorption/desorption isotherm curves of iron oxide fibers using slow drying (**a**) and fast drying (**b**). Fe:Mn = 1:1 fibers by slow drying (**c**) and fast drying (**d**).

Figure 7 presents the nitrogen adsorption/desorption isotherms for iron oxide and Fe:Mn = 1:1 fibers produced through slow and fast drying methods. Table 2 outlines the BET-specific surface area, the average pore diameter, and the BJH pore volume of the fibers.

Based on the IUPAC classification [27], the fibers exhibit type IV isotherms and type H1 hysteresis loops, typical of mesoporous materials. The dried fibers (in the synthesis step) display N_2_ adsorption in the low-pressure region (P/P0 < 0.2), indicating a mesoporous structure, which is particularly significant for the Fe:Mn = 1:1 sample [28] (as shown in Figure 4d). This behavior reflects the presence of non-uniform pores that vary in size and shape, as observed in the MET images (Figure 5c,d,g,h).

The presence of mesopores can be attributed to channels formed between two or more grains (fibers particles) and also to the coalescence of fibers (interfibrous space) [26]. Additionally, the rapid drying caused an increase in the surface area of iron oxide and Fe:Mn = 1:1 fibers by approximately 2.3× and 2.5×, respectively (see Table 2), due to an increase in porosity. It is worth noting that despite the larger mean diameters of fibers produced by rapid drying, the generated porosity was crucial for increasing the surface area. 

The BET-specific surface areas obtained with the fast-drying method are superior to fibers prepared by electrospinning, a widely used technique. Bhagwan and collaborators [26] obtained CdMn_2_O_4_ nanofibers with a BET surface area of 2 m^2^/g. Han and collaborators [29] obtained SmFeO_3_ fibers with a BET surface area of 2.87 m^2^/g. Furthermore, articles involving materials for energy storage presented similar or less specific areas. Na_4_Fe_3_(PO_4_)_2_(P_2_O_7_) nanofibers exhibited a BET surface area of 35.24 m^2^/g [30]. Mesoporous Mg_0.9_Zn_0.1_Fe_2−x_Mn_x_O_4_ nanoparticles showed a BET surface area from 7.36 m^2^/g to 14.04 m^2^/g [31]. These results indicate that the fast drying used in the SBS process can produce promising mesoporous fibers for energy storage and catalysis applications due to the high surface area.

## 4. Conclusions

This work prepared pure α-Fe_2_O_3_ fibers and FeMnO_3_/α-Fe_2_O_3_ fibers using two drying modes during Solution Blow Spinning followed by heat treatment at 800 °C. The fast-drying SBS system produced fibers, preserving the fibrillar structures of systems containing low-volatility solvents and hygroscopic precursors. TEM transmission electron microscopy confirmed the surface roughness and densification of the fibers obtained by slow drying. In addition, rapid drying yielded irregular porosity and low fiber densification. Rapid drying favored the production of higher surface area fibers, as depicted in N_2_ adsorption/desorption analysis, and allowed the successful use of low volatility solvents in the processing of Fe:Mn fibers by SBS.

## Figures and Tables

**Table 1:** *p*-values calculated from the Mann–Whitney test of fiber diameter distributions.

	Iron Oxide (F.D *.)	Fe:Mn = 1:1 (F.D *.)	Iron Oxide (S.D **.)	Fe:Mn = 1:1 (S.D **.)
Iron oxide (S.D **.)	0.012	<0.001	-	<0.001
Fe:Mn = 1:1 (S.D **.)	<0.001	<0.001	<0.001	-
Iron oxide (F.D *.)	-	0.921	0.012	<0.001
Fe:Mn = 1:1 (F.D *.)	0.921	-	<0.001	<0.001

* F.D.—Fast drying, ** S.D.—Slow drying.

**Table 2:** BET surface area, pore diameter, and pore volume of iron oxide and Fe:Mn = 1:1 fibers.

	Sample			
	Slow Drying		Fast Drying	
	Iron Oxide	Fe:Mn = 1:1	Iron Oxide	Fe:Mn = 1:1
Specific surface area (m^2^/g)	14.6	28.7	34.9	73.3
Average pore diameter (nm)	3.1	2.9	2.7	2.9
Pore volume (cm^3^/g)	0.032	0.038	0.015	0.031

## Data Availability

The data presented in this study are available on request from the corresponding author, Romualdo Rodrigues Menezes.
